# Upon the Utility of Cold Affusion in Cases of Poisoning by Hydrocyanic Acid

**Published:** 1829-05

**Authors:** E. F. Gustave Herbst


### HYDROCYANIC ACID.

### *Upon the Utility of Cold Affusion in Cases of Poisoning by Hydrocyanic Acid.* By Dr. E. F. Gustave Herbst.

Hydrocyanic acid is the most violent of all known poisons. Numerous experiments have been made to discover its mode of action; from which it appears to act principally on the nervous system; the symptoms which it produces on the lower animals, when given in pretty large doses, leaving little room to doubt but that its first effect is excessive excitation of the nervous system, whose powers it rapidly exhausts. Hence it is speedily followed by paralysis, muscular flaccidity, insensibility, &c. which are the forerunners of approaching death. The means, therefore, proper to counteract the effects of this poison are such as are calculated to restore the lost sensibility of the nervous system.

Several antidotes have been proposed, which, though not devoid of utility, have not quite produced the desired effect: up to the present time, Liquor Ammoniæ, which possesses such great influence over divers poisons, has been also much employed against this. In fact, it diminishes the effects of the prussic acid, especially when given immediately after the poison, and where the dose of the acid would not kill the animal, if quite left to itself: but should some time be allowed to pass, and the quantity of acid be sufficient to destroy the animal, then the effect of the ammonia is less powerful and less certain, and for the most part finally unavailing. Besides, when the aqua ammonia is diluted with water enough to moderate its caustic qualities, it becomes in proportion less powerful; and, though more efficacious, it is also more dangerous in its concentrated state. If the liquor ammoniæ is given undiluted after a dose of prussic acid sufficient to cause death, the following phenomena are usually observed: The animal, from a state of violent convulsions, suddenly recovers the use of his muscles, frisks, takes a few steps, and, again falling down, reassumes his former condition; but the spasms are not so violent, and sometimes entirely cease. The animal remains in this state until excited by another dose of the ammonia, when it moves a few steps, and then again becomes torpid. Sometimes, if the doses are repeated, it recovers the use of its muscles for a longer time, and by degrees is quite restored: but generally, after the first doses, the ammonia produces no lasting benefit; for, though life is sustained for a short time, it is not ultimately preserved by its use. Nor should it be forgotten that the difficulty of deglutition, which is one of the consequences of an overdose of prussic acid, diminishes the value of those antidotes which must be exhibited internally.

When hydrocyanic acid is given in doses which would not, if uncounteracted, prove fatal, cold affusion, only two or three times repeated, is sufficient to neutralize its effects. But, if the dose has been more considerable, the affusion must be copious and frequent; yet its efficacy will chiefly depend on its prompt employment. Cold affusion can be especially relied upon when used immediately after the acid, or during the convulsive stage, even as long as the eyes are insensible and immoveably fixed, the extremities stiff and extended, and the head thrown back. To this state there succeeds general relaxation of the muscles, the respiration becoming gradually imperceptible, the pulse slow, weak, and scarcely to be felt, and in an instant afterwards death supervenes. Even at this period of paralysis, cold water recalls the vitality ready to vanish. The muscles again contract and become hard, the extremities rigid, and every thing successively resumes its natural state. The relation of a few experiments will serve to confirm these assertions.

Dr. Herbst gave to a middling-sized mastiff, three years old, in divided doses, fourteen drops of prussic acid. The dog after some time fell down, and the effects of the poison were strongly manifested. The cold affusion would, says Dr. H., have speedily restored him; but, during the convulsive stage, about twenty drops of liquor ammoniæ were poured down its throat. He immediately sprung up, ran a few steps, and soon fell down again. The muscles were no longer so forcibly contracted as at first. He took, by degrees, one drachm and a half of the liquor ammoniæ, without deriving any apparent benefit in the absolute; though, after each dose, he leaped and ran a little. Another dose of fourteen drops of the acid reproduced most violent tetanus, which was not in the least assuaged by half a drachm of the ammonia. In a few minutes the dog was dead. His tongue and throat were excoriated, and covered with blood.

Dr. H. gave to a small dog, eighteen months old, a considerable quantity of the same acid. The usual symptoms supervened, which (according to Dr. H.) the cold affusion would have easily removed.† He poured down its throat half a drachm of liquor ammoniæ, and the dog immediately rose, sprung forwards, and again fell down; the violent tetanus had ceased, the extremities were no longer stiff. But fresh doses of the ammonia, to the amount of one drachm, were of no avail. The dog was allowed to remain in this state half an hour, when, the respiration becoming gradually weaker, he appeared to be nearly dead. Its head and back were then inundated with water; and his state was thus in some minutes so greatly improved, that he was able to walk with difficulty. The cold affusion was then intermitted, in order to see if he would recover without further aid. At the end of twelve hours the dog was still living, much exhausted, and did not eat; was found dead eighteen hours after. The throat, which had bled a great deal, was much excoriated.

He gave to a puppy of three months eight drops of prussic acid. It was immediately affected in the usual way, and fell down. Dr. H. gave it half a drachm of liquor ammoniæ, in a small quantity of water. The fluid for some time remained in its throat, but, when the animal began to revive and to move about, the greater part was swallowed. The dog soon relapsed into its former state: the muscles became flaccid, and its prostration was so great that fresh doses of ammonia were not even swallowed. The dog died five minutes from the commencement of the experiment. At the dissection, which immediately took place, the general irritability of the muscles appeared to be very great; the heart continued beating for ten minutes;* the venæ cavæ were full of blood, but the brain was not remarkably red.

These experiments prove that ammonia, when used as an antidote to prussic acid, does not always produce the desired effect; for, even when the violent contraction of the muscles was diminished, and a sudden brisk excitation of the vital powers effected, the prostration became greater, and death ensued, even in those cases in which the ammonia was administered under the most favorable circumstances.

Dr. Herbst gave six grains of hydrocyanic acid to a small adult dog, which caused him immediately to utter a plaintive cry, fall down, stiffen his legs, and after some minutes appear dead. His head, back, and whole body were affused with cold water: the respiration was reestablished, and in a few minutes the dog attempted to rise; in fifteen, he could walk; and in half an hour was quite restored.

To a strong bitch, about five years old, Dr. H. gave eight grains of the acid: it was in a few moments seized as the

* The knowledge of this singular fact, in 1809, induced Professor Brera, of Padua, to employ the hydrocyanic acid in inflammation of the lungs.—Editors.

last, and was about to fall, when cold water was poured upon its head; by which means it was, after a few minutes, quite recovered. It took several small doses, each of which respectively produced a sort of stupor; but this was always removed by the cold affusion. A quarter of an hour after, the animal vomited a great deal of a sort of white thick mucus, and it foamed; but these symptoms ceased in about an hour and a half, when it had taken its ordinary food.

The next morning this bitch again took eight grains of the acid, and immediately fell down. Violent opisthotonos, difficult respiration, and gradual prostration, seemed to announce certain death. Cold water was immediately poured upon its head. In one minute, the breathing having become more free, the dog raised its head, looked about with an expression of astonishment, but remained lying down. After a continuation of the affusion, it rose, and walked with difficulty; and an hour afterwards it was completely restored.

The same experiment, with the same result, was made upon several other dogs; but, in order to prove beyond a doubt the efficacy of cold affusion in counteracting the effects of prussic acid, Dr. H. instituted the two following experiments:

He chose two young dogs, of the same age and size, which had been similarly nourished: he gave four drops of the acid to one of them. After the dose, the animal became quiet, staggered; but soon recovered. A fresh dose of eight drops made it fall, cry out piteously, and vomit a sort of mucus. Although much exhausted, it recovered once more, the opisthotonos ceased; but, after taking four more drops, it rapidly sank, and was dead within four minutes from the exhibition of the first dose.

To the second dog Dr. H. gave sixteen drops in one dose. At first it turned round, then fell down, incapable of motion or feeling. The head was thrown backwards, the legs extended. In thirty seconds the respiration was imperceptible, the beating of the heart nearly so. Dr. H. hastened to use the cold affusion, for the muscles were already relaxed, and the animal appeared to be dead. The The cold water at first seemed to effect no change: the first sign of life was the opisthotonos; the relaxed extremities contracted again; whilst the dog uttered a feeble plaintive cry, which soon became stronger. The rigidity of the trunk continued a long time. Cold water was poured all over the animal, who continued to cry, and its body, which was stiff and hard, seemed painful when touched. The

Cruelty to Animals affusion was kept up for fifteen minutes; during which the respiration became stronger; but it became alternately stronger or weaker, as often as the affusion was interrupted or renewed. The same day the dog was completely restored, ran about, barked, and eat as though nothing had occurred.

Dr. B. injected into the left external jugular vein of a dog nine drops of hydrocyanic acid; five minutes after, twelve drops; and after five minutes more, fifteen drops. There was no alteration in the dog, except that his heart beat very feebly, rapidly, and irregularly. Then twelve grains (about twenty-eight drops) were injected at once, and produced great disturbance, involuntary discharge of the excrements, violent tetanus, &c. and soon afterwards general prostration, and almost imperceptible respiration. The cold affusion on the head immediately made the dog rise; its continuation removed the paralysis of the posterior extremities. In a quarter of an hour the dog had quite recovered.

Three days afterwards, Dr. H. injected into the right external jugular of the same dog fifty drops of hydrocyanic acid at once. This time the tetanus was even more violent; however, the cold affusion was not employed till the whole body was in the paralytic stage, excepting the muscles of the eyes, which were still in a state of convulsive contraction. The same result was obtained in this instance; for, in four hours afterwards, the dog appeared as if nothing had happened to him.

Barring a few failures, arising from accidental circumstances, the same favorable results occurred in all the other cases in which the poison was injected into the veins, wounds, nostrils, or eyes. But, when the acid was injected in great quantity, the effects were so sudden that, after the tetanus was become general, there was scarcely time to commence the affusion before death should supervene.

These experiments demonstrate that the hydrocyanic acid, when administered even in doses which would be more than enough to cause death, may be promptly neutralized by cold affusion upon the head, the back, and the whole body, by which it loses all its deleterious properties.*

* Archiv für Anatomie und Physiologie, by J. F. Meckel.

